# Cases of Lightning Strikes during Mountain-Sports Activities: An Analysis of Emergencies from the Swiss Alps

**DOI:** 10.3390/ijerph19073954

**Published:** 2022-03-26

**Authors:** Benedikt Gasser

**Affiliations:** A Division of Sports and Exercise Medicine, Department of Sport, Exercise and Health, University of Basel, 4052 Basel, Switzerland; benediktandreas.gasser@unibas.ch; Tel.: +41-78-817-07-11

**Keywords:** emergency medicine, electric injuries, occurrence of lightning strikes, resuscitation, cause of death

## Abstract

Background: Lightning strikes are a risk during mountain-sport activities. Yet little is known about the prevalence of injuries related to lightning strikes during mountain hiking, backcountry skiing, or high-altitude mountaineering. This study therefore examined the occurrence and characteristics of lightning-strike-related emergencies during mountain-sport activities in the Swiss Alps. Methods: We analyzed 11,221 alpine emergencies during mountain hiking, 4687 during high-altitude mountaineering, and 3044 during backcountry skiing in the observational period from 2009 to 2020. Identified cases were analyzed in detail regarding age, sex, the time of occurrence, altitude, location, the severity of the injury as quantified by its NACA Score (National Advisory Committee for Aeronautics Score), and injury pattern. Results: We found no cases related to backcountry skiing. Eight cases of lightning strikes during mountain hiking (four female and four male) were identified. The mean age was 32.5 ± 17.5 years, the mean NACA Score was 2.5 ± 1.9, and the mean altitude was 1883.8 ± 425.7 m. None of these cases were fatal, and only one victim was seriously injured. Fifteen cases were identified during high-altitude mountaineering (four female and 11 male). The mean age was 38.7 ± 5.2 years, the mean NACA Score was 3.1 ± 2.5, and the mean altitude was 3486.4 ± 614.3 m. Two lightning strikes were fatal. In these two cases, rope partners were injured by a lightning strike (NACA Score = 4). Most cases were on relatively exposed terrain, such as the Matterhorn Hörnligrat or the Eiger Mittellegigrat. Discussion: The typical victims were 30–40-year-old men. It is possible that the lightning strikes are a consequence of a lower risk aversion among these alpinists, which is be supported by the fact that most of the events occurred on famous mountains such as the Matterhorn or Eiger. Furthermore, since most of the locations were on relatively exposed terrain where one could not quickly find shelter, we recommend careful tour planning with serious consultation of the weather forecast and the likelihood of thunderstorms before climbing exposed sections to prevent emergencies related to lightning strikes.

## 1. Introduction

Mountaineering in its various forms, including backcountry skiing, high-altitude mountaineering, and mountain hiking, is being practiced by an increasing number of people [1,2,3]. It has been estimated that around 350,000 backcountry skiers, 150,000 high-altitude mountaineers, and two million mountain hikers are active per year in the Swiss Alps [4]. These activities entail serious risks that can potentially lead to emergencies. Avalanches, falls, frostbite, rock falls, or becoming lost are known dangers that go hand in hand with these mountaineering activities [5,6,7]. In comparison to these frequent causes of emergencies, less is known about lighting strikes [8]. While the general risk of being struck by lightning is low, the risk is increased in the mountains due to one’s proximity to the atmosphere [8,9,10,11]. Lightning strikes are electrical discharges in the earth’s atmosphere [12,13,14,15]. Depending on the weather conditions, electric tensions of more than 100,000,000 volts can occur [12,13,14,15]. A lightning discharge lasts about 0.1–0.3 s, with maximum duration up to 1–2 s [12,13,14,15]. It is accompanied by currents of up to 400,000 amperes [12,13]. The air in the lightning channel heats up to around 25,000–30,000 °C [13]. In doing so, it expands explosively, causing the well-known acoustic effects of a thunderstorm [12,13,14,15]. More than 100 of these natural events occur each second worldwide, whereby most of them are intracloud flashes and only around ten percent cloud to ground flashes [8,9,15]. For Switzerland, around 60,000–80,000 lightnings (intracloud and cloud-to-ground) are reported [16,17]. Regions with an increased likelihood are along the jura, the north side of the alps and most prominent in the south of canton Tessin [16]. The effects of lightning strikes are manifold. A direct lightning strike is the most dangerous [11,18,19]. If a person is struck directly by lightning, the voltage in the body can increase up to 100,000 volts, which is normally fatal [9,12].

Furthermore, people who are under a tree or near an object struck by lightning are likewise at risk through a flashover [12]. The flashover phenomenon is observed in all modes where energy is transferred [12]. The vast majority of the voltage does not flow through victims but remains on the surface of the body [8,12,18]. Based on the current evidence, this effect accounts for why the majority of people hit by a lightning strike survive [8,12,18]. If death immediately follows the event, it is usually due to a lethal cardiac arrhythmia with ventricular fibrillation and subsequent or immediate asystole [18]. If the lightning strike is initially survived, myocardial infarction, shock from burns, secondary renal failure, and trauma-related cerebral hemorrhage may occur [18]. A lightning strike can cause numerous different lesions in survivors [18]. Injuries result from the electric energy, the high temperature, or the explosive force of the blast wave [18,19,20,21]. Injuries to the skin (burns) and the heart and affections of the central nervous system, ears, and eyes are most commonly seen in survivors [9,12,19,20,21,22,23,24,25]. In mountains with rocky subsoil, the lightning current can spread across long distances [12]. When touching a rock, part of the lightning can flow over the body, and cases have been described where the mountaineer was even thrown away [12].

However, emergencies due to lightning strikes are rarely discussed; they are mainly known from the stories of trees standing alone on an alpine meadow in which lightning has left its mark [20]. Nevertheless, activities in the mountains and the associated proximity to the atmosphere expose alpinists to a considerable risk. In the event of an approaching thunderstorm, protection should be sought as soon as possible [12]. Reasonable possibilities include inside huts with a lightning rod or at the foot of a rock face, where one should ideally keep a certain distance of two to three meters from the rock face [12]. Despite these known preventative measures, little is known about the likelihood of lightning strikes in the Swiss Alps during mountain-sport activities. This study therefore analyzed lightning strikes during mountain hiking, high-altitude mountaineering, and backcountry skiing in the Swiss Alps with regard to their occurrence, mechanism, injury pattern, and likelihood as compared to other causes of mountain emergencies [26].

## 2. Materials and Methods

### 2.1. Study Population

This study used data of the Swiss Alpine Club (SAC) central registry. The registry contains emergencies that occurred during high-altitude mountaineering (from 2009 to 2020), backcountry skiing, and mountain hiking (both from 2009 to 2018). The data were collected by the Swiss Air Rescue Service (REGA), Air Glaciers Lauterbrunnen, Air Glaciers Sanenland, Register SAC, KWRO (Kantonale Walliser Rettungsorganisation), Snow and Avalanche Research Institute Davos, and the cantonal police. The term “mountain emergency” covers all events involving mountaineers claiming the help of mountain rescue services or those that are affected by subjective and objective mountain hazards [6,7]. This also applies to illnesses and evacuations of uninjured mountaineers. Each case recorded in the database included information about the emergency number used, date, rescue organization, event, place, canton, activity, National Advisory Committee for Aeronautics Score (NACA Score; see Supplementary Table S1), nationality, age, sex, place of residence, coordinates, and a short report [27,28]. For the present study, all cases of lightning strikes were filtered and analyzed in detail.

### 2.2. Data Preparation

Data were classified according to the cause of the mountain emergency. In the 12-year period of 2009–2020, a total of 4687 alpinists (1027 female and 3660 male) were rescued by the mountain rescue service in the Swiss Alps while high-altitude mountaineering. Furthermore, 11,125 mountain hikers (4933 female and 6192 male) and 3044 (953 female and 2091 male) backcountry skiers were rescued by the mountain rescue service in the Swiss Alps between 2009 and 2018. The classification by discipline high-altitude mountaineering versus mountain hiking versus backcountry skiing was made by the professional emergency services. All cases were subsequently analyzed in detail regarding age, sex, time of occurrence, severity of an event quantified with a NACA Score (National Advisory Committee for Aeronautics Score), location and injury pattern [29,30].

### 2.3. Statistical Analyses

Descriptive statistics were calculated for age and NACA Scores. A Mann-Whitney U test was used to analyze differences in the severity of injuries between high-altitude mountaineering and mountain hiking. The same procedure was performed to analyze between-sex differences in NACA Scores. A linear regression with calculating coefficient of determination (R*^2^*) was performed in order to detect a potential alteration of the number of cases over time. Analyses were performed with Microsoft Excel 2016 (Microsoft Inc., Redmond, WA, USA) and SPSS statistics Version 27 (Armonk, New York, NY, USA).

## 3. Results

**Figure 1:**
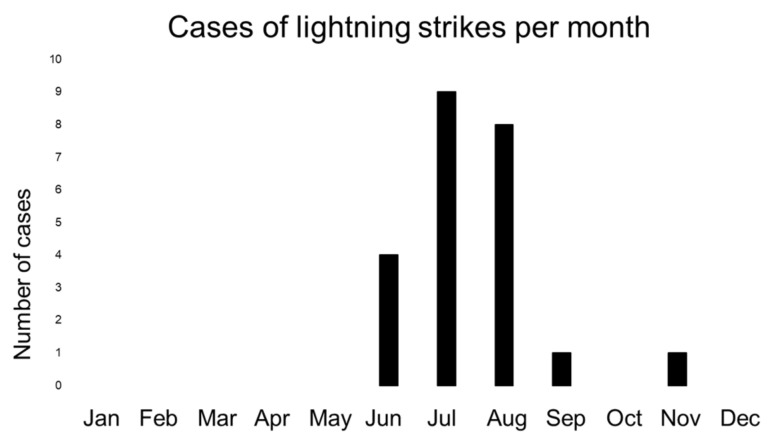
Occurrence of lighting-strike-related emergencies in the Swiss Alps stratified by months for all cases (high-altitude mountaineering and mountain hiking).

**Figure 2:**
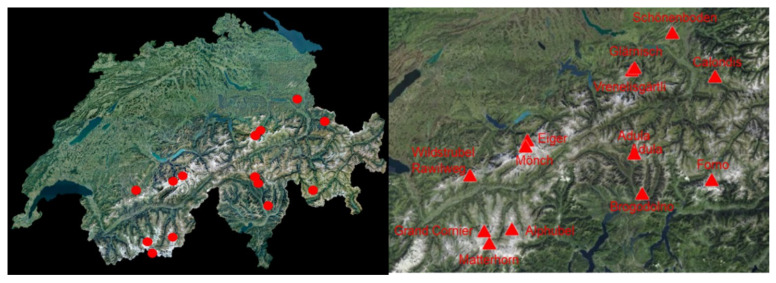
Geographic locations of lightning strikes in the Swiss Alps (map from Swisstopo [31]). Interestingly, cases are along the main massive of the alps.

**Figure 3:**
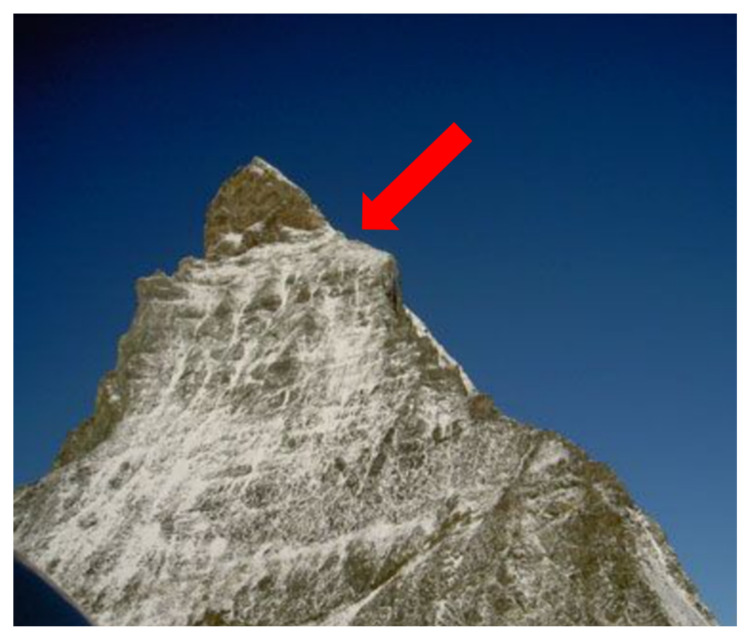
The Hörnligrat on Matterhorn. Lightning strike-related emergency were identified close to the shoulder (red arrow). Seeking shelter on a ridge is sometimes simply not possible.

Out of a total of 11,125 emergencies during mountain hiking, there were eight cases (four male, four female) of lightning strikes (0.07%) in the observational period. For high-altitude mountaineering, 15 cases of lightning strikes (11 male, four female) were detected from a total of 4687 emergencies (0.32%). No cases were recorded during backcountry skiing in the observational period. The majority of all cases occurred in the summer except one case in September and one case in the beginning of November (Figure 1). Figure 2 gives an overview of the geographical location of the emergencies. Two hot spots were detected: in the Valais region and in the Jungfrau area. Furthermore, as it is the southernmost part of Switzerland, some cases in Tessin in line with other indicators suggesting that the number of thunderstorm days and lightning strikes per km^2^ increases from north to south with the highest occurrence in the Canton Tessin (Figure 3) [12,16,17].

Regarding the eight cases during mountain hiking, the mean age was 32.5 ± 17.5 years and the mean NACA Score as indicator of the severity of an injury was 2.5 ± 1.9 (Supplementary Table S1). None of these cases were fatal. One case needed cardiopulmonary resuscitation (NACA Score = 6), which was a severe cardiac arrhythmia. All of the others had a NACA Score of only 3 or less, which indicates a non-life-threatening condition. Like most of the cases while high-altitude mountaineering (next paragraph), these cases consisted of only moderate injuries (for example fracture of a finger bone, moderate cuts, dehydration or even a femur fracture, see Supplementary Table S1) with signs of paralysis but no cardiovascular symptoms. The mean altitude of the emergencies was 1883.8 ± 425.7 m. The eight cases during mountain hiking were detected close to the Capanna Brogodone, Capanna Adula, Vorder Glärnisch, Schoenenboden Wildhaus, on the Rawilweg in the Wildstrubel area, and on the Calondis (Figure 2).

While high-altitude mountaineering the mean age of victims was 38.7 ± 5.2 years. The mean NACA Score as indicator of the severity of an injury was 3.1 ± 2.5 (Supplementary Table S1). Two lightning strikes were fatal, and in these cases other members of the rope team were also seriously injured by flashovers with a NACA Score of 4. Moderate injuries of the extremities were identified in six cases with signs of paralysis in the most-exposed extremity (hand or foot) but no obvious cardiovascular symptoms. A loss of consciousness with cardiac arrest was detected in five cases. In two cases, no detailed information concerning clinical symptoms was identified in the case reports. The mean altitude was 3486.4 ± 614.3 m. The cases were mainly on popular mountains higher than 4000 m such as the Matterhorn, Grand Cornier, Mönch, Eiger, or Alphubel. Only two cases were below 3000 m on the Vrenelisgärtli and at the Fornohütte (Figure 2). Detailed analysis revealed that most cases were relatively exposed, such as on the Mittellegigrat of the Eiger or on the Hörnligrat of the Matterhorn (Figure 3).

Concerning the nationality of the emergency victims, of the high-altitude mountaineers, nine victims were from Switzerland, five were from Germany, two were from Italy, five were from the Czech Republic, one was from the United States, and one was from Great Britain. Thus, about seventy percent (69.5%) were from countries in which the Alps are located: Switzerland, Italy, and Germany. Keeping the small n in mind limiting the clinical significance of the statements, neither the severity of the injuries (*p* = 0.097) nor the age of the victims (*p* = 0.271) was significantly different between mountain hikers and high-altitude mountaineers. In addition, NACA Scores (*p* = 0.920) and age (*p* = 0.098) were not significantly different between the sexes in both mountain hikers and mountaineers. A linear regression revealed no significant alteration of the number of cases over time for high-altitude mountaineering (number of cases = 0.0385 × year−76.397, R^2^ = 0.0057) and for mountain hiking (number of cases = 0.007 × year−13.4, R^2^ = 0.0006) indicating no alterations of emergencies over time.

## 4. Discussion

This study aimed to analyze emergencies related to lightning strikes during mountain-sport activities in the Swiss Alps. Out of a total of 11,125 emergencies during mountain hiking, there were eight cases (none fatal) of lightning strikes (0.07%) in the observational period. This translates to a prevalence of 0.8 cases per year. For mountaineering, 15 cases of lightning strikes (two fatal) were detected from a total of 4687 emergencies (0.32%), yielding 1.3 cases per year. No cases were recorded during backcountry skiing in the observational period. To conclude, the death rate was low with 8.7% (two out of 23 cases), which is in line with others’ findings [13]. In principle, these values seem valid as since the 1940′s the ten-year average of fatal lightning events decreased from around 5–10 cases in the 1940′s to zero to one in the years after 2000 [32]. These findings also correspond to the detected injury pattern (not life-threatening injuries in most cases due to a flashover) in most of the identified emergencies. The overall prevalence of being struck by lightning is very low, in the same range as, for example, being bitten by a snake while hiking [33,34]. This correlates with reports from Germany [13]. While around 50–100 people died from lightning strikes per year in Germany 50 years ago, the number of fatally injured people has steadily decreased to three to seven deaths per year since the millennium, which is in line with our findings [13].

In our analyses, the typical victim was a 30–40-year-old male, which is in line with previous findings in the same context [24]. Considering the higher physical fitness of this age group as compared to, for example, seniors, alpinists in this age range might have a lower risk aversion [34]. Furthermore, a higher activity level than in older individuals during suboptimal weather conditions might further increase their likelihood of being struck by lightning [34]. Potentially supporting the argument, as most cases were on popular tours undoubtful often absolved, it is in addition likely to suggest that subjects try to climb these mountains without having the skills needed not estimating (meteorological) risks in an adequate manner.

Analyses further revealed that a considerable number of events were on highly exposed terrains, such as the Mittelleggigrat of the Eiger or the Hörnligrat of the Matterhorn (Figure 3). This is in line with the publicly available information, which stated that the alpinists struck by lightning were often on exposed terrains such as a ridge [35]. The pattern often seems to be the same [35]. A storm moves in very quickly, and alpinists hurry to descend from an exposed ridge [35]. Unfortunately, it is impossible for them to descend quickly enough, and lightning strikes a part of an extremity [35]. To conclude, on exposed terrain on a ridge, quickly seeking shelter during sudden weather changes is sometimes simply impossible. Prevention thus has to be performed in the stage of tour planning with careful consideration of the weather forecast. The influence of the weather is further supported by the trivial fact that the vast majority of cases occurred in the summer months, when mountaineering activity is at its highest; this also explains why no case was detected while backcountry skiing, which is mainly practiced in winter [8,36]. Concerning the validity of the findings, a study on the Austrian Alps also reported that lightning strikes are mainly present in summer months [8]. In that study, a total of 64 cases were detected over a ten-year observation period from 2005 to 2015 [8]. Four people died, which yields a survival rate of 93.8% [8], which is similar to the survival rate of 91.3% in our data. The slightly higher mortality in the Swiss Alps might be due to the simple fact that mountains are in general higher in Switzerland than in Austria, which results in more severe events. In addition, these differences could be associated to different meteorological conditions between Swiss and Austria [17]. However, whether this is actually the case would have to be elucidated in the future.

The validity of the study is also corroborated by an analysis on the United States [37] that reported around 20 deaths related to lightning strikes per year; a majority of them were associated with outdoor-recreational activities. The reported values of mortality were comparably low and estimated to be between 10% and 30% [9,38]. In the study on the Austrian Alps, 64 people were struck by lightning while recreationally active, most while hiking (*n* = 55), a few while hunting, and only one while doing occupational forestry [8]. Interestingly, the prevalence of emergencies related to lightning strikes seems higher in Austria than in Switzerland [8]. These differences might be explained by the size of their geographical areas. The Swiss Alps cover around 24,850 km^2^, which is only 45% of the area covered by the Austrian Alps: 54,600 km^2^ [39,40]. It is thus tempting to assume that although only cases during mountain-sport activities were analyzed, this study potentially covered a substantial share of emergencies related to lightning strikes in Switzerland. This is directly related to a limitation of the findings: only events from the registry were included in the analysis. It is probably seldom the case that someone is struck by lightning and does not contact official emergency services, but it would be informative to know how frequently alpinists are nearly struck by lightning, which is, of course, not captured by the analysis.

To summarize, alpine emergencies involving lightning strikes are rare. The typical victim is a 30–40-year-old male on exposed terrain in the summer months. Prevention seems mainly possible during tour planning, for example, in the SAC hut on the day before going on an exposed ridge. The most important factor is carefully consulting the weather forecast in order to minimize the likelihood of being caught by a thunderstorm on the mountain. To stay, for example, an SAC hut and not undertake the route during unclear weather conditions is simply sometimes the wiser choice than an attempt to reach the peak. If something happens despite these recommendations, the emergency management of apparently lifeless persons should take priority in accidents involving victims due to lightning strikes. The chances of successful cardiopulmonary resuscitation are relatively good in comparison to other causes of life-threatening emergencies [13,17,41,42,43]. The rule of thumb, to pay first attention to subjects with recognizable signs of living is in this special situation misleading as the chance of cardiac resuscitation of victims due to lightning strikes is considered as high [13,17,41,42,43].

## Supplementary Materials

The following supporting information can be downloaded at: https://www.mdpi.com/article/10.3390/ijerph19073954/s1, Supplementary Table S1—Description of National Advisory Committee for Aeronautics Score (NACA-Score).

## Abbreviations

SAC	Swiss Alpine Club
NACA-Score	National Advisory Committee for Aeronautics Score
REGA	Swiss Air Rescue Service-Schweizerische Rettungsflugwacht
