# Supplementary material for: Cases of Lightning Strikes during Mountain-Sports Activities: An Analysis of Emergencies from the Swiss Alps
Source: Int J Environ Res Public Health. 2022 Mar 26;19(7):3954. doi: 10.3390/ijerph19073954 (PMC8998020; doi:10.3390/ijerph19073954)
Supplement: Supplementary file 1 [file ijerph-19-03954-s001.zip › ijerph-1607607-supplementary.pdf]

Supplementary Table S1. Description of National Advisory Committee for Aeronautics Score (NACA-Score) [23,24].

NACA 0	No injury or disease.
NACA I	Minor disturbance. No medical intervention is required (e.g., slight abrasion).
NACA II	Slight to moderate disturbance. Outpatient medical investigation but usually no emergency medical measures necessary (e.g., fracture of a finger bone, moderate cuts, dehydration).
NACA III	Moderate to severe but not life-threatening disorder. Stationary treatment required, often emergency medical measures on the site (e.g., femur fracture, milder stroke, smoke inhalation).
NACA IV	Serious incident where rapid development into a life-threatening condition cannot be excluded. In the majority of cases, emergency medical care is required (e.g., vertebral injury with neurological deficit, severe asthma attack, drug poisoning).
NACA V	Acute danger (e.g., third grade skull or brain trauma or severe heart attack).
NACA VI	Respiratory and or cardiac arrest.
NACA VII	Death.
